# The Mechanism of Valence-Space Metaphors: ERP Evidence for Affective Word Processing

**DOI:** 10.1371/journal.pone.0099479

**Published:** 2014-06-12

**Authors:** Jiushu Xie, Ruiming Wang, Song Chang

**Affiliations:** Center for Studies of Psychological Application, School of Psychology, South China Normal University, Guangzhou, Guangdong Province, China; Centre de Neuroscience Cognitive, France

## Abstract

Embodied cognition contends that the representation and processing of concepts involve perceptual, somatosensory, motoric, and other physical re-experiencing information. In this view, affective concepts are also grounded in physical information. For instance, people often say “feeling down” or “cheer up” in daily life. These phrases use spatial information to understand affective concepts. This process is referred to as valence-space metaphor. Valence-space metaphors refer to the employment of spatial information (lower/higher space) to elaborate affective concepts (negative/positive concepts). Previous studies have demonstrated that processing affective words affects performance on a spatial detection task. However, the mechanism(s) behind this effect remain unclear. In the current study, we hypothesized that processing affective words might produce spatial information. Consequently, spatial information would affect the following spatial cue detection/discrimination task. In Experiment 1, participants were asked to remember an affective word. Then, they completed a spatial cue detection task while event-related potentials were recorded. The results indicated that the top cues induced enhanced amplitude of P200 component while participants kept positive words relative to negative words in mind. On the contrary, the bottom cues induced enhanced P200 amplitudes while participants kept negative words relative to positive words in mind. In Experiment 2, we conducted a behavioral experiment that employed a similar paradigm to Experiment 1, but used arrows instead of dots to test the attentional nature of the valence-space metaphor. We found a similar facilitation effect as found in Experiment 1. Positive words facilitated the discrimination of upper arrows, whereas negative words facilitated the discrimination of lower arrows. In summary, affective words might activate spatial information and cause participants to allocate their attention to corresponding locations. Valence-space metaphors might be grounded in attention allocation.

## Introduction

Embodied cognition holds that affective words are grounded in the physical world. Processing affective words affects spatial detection tasks. For instance, positive words promote the detection of upper stimuli and negative words promote the detection of lower stimuli, referred to as the metaphorical congruency effect [1]. However, the mechanism by which affective words induce this effect is unclear. It is plausible that processing affective words activates spatial information and that spatial information shifts participants’ attention into corresponding locations. We will test this hypothesis in the present experiment.

Concepts are vital to human cognition, as they allow for generalization of useful environmental information and individual experiences. According to the cognitive economy principle, cognitive load is decreased by such abstractions [2]. These abstractions are the basis of analysis, integration, and summation in the cognitive system. In sum, concepts are the glue of human cognition, as they hold the human mental world together [3].

Embodied cognition states that cognition is grounded in bodily states, which constitute the basis of sense, perception, behavior, introspection, and so forth. Bodily states refer to current bodily states or experiences of previous bodily states [4], [5]. In contrast to traditional cognitive theories, embodied cognition holds that sensorimotor information is vital to human cognition. For instance, when people comprehend language, specific modalities that relate to the words are simultaneously activated, such as the visual, auditory, or tactile sense [5]. Further, when people process language, sensorimotor representations are partially activated or reconstructed according to different processing situations, which could not be predicted by traditional theories, such as the semantic network [6]. For instance, “skyscraper” and “airplane” are related to spatial information (top space) and processing of these two words activates representations of upper spatial information. However, neither word has a direct link with the top space in the semantic network. In addition, because cognition relies on individual experiences and different people have varying experiences and physical bodies, cognitive processing varies between individuals [4]. In short, embodied cognition holds that representation and processing of knowledge always involves perceptual, somatosensory, and motoric re-experiencing of information [7].

Several studies have provided evidence for embodied cognition. For instance, studies have found that spatial information participates in abstract conceptual processing. Smith [8] allowed 30-month old infants to learn a new conceptual tag, “wug.” The experimenter provided a novel prototype and taught infants that it was a “wug.” Infants then learned this new concept in four different ways. First, infants barely observed the experimenter move the prototype vertically or horizontally. Second, infants moved the prototype vertically or horizontally by themselves. Third, infants clearly observed the experimenter move the prototype back and forth. Fourth, infants directly determined which exemplar was the “wug” after learning the prototype. Infants believed the wide exemplar was the “wug,” when they moved the prototype or saw the experimenter move the prototype horizontally. Similarly, they believed the long exemplar was the “wug,” when they moved the prototype or saw the experimenter move the prototype vertically. Thus, it appears that conceptual learning is grounded in spatial and motion information.

Conceptual representations are also grounded in bodily states. In conceptual processing, participants judged the relationship between two words (related vs. unrelated). The word pairs contained location information, and these words were represented according to or opposite their implicit meanings. For instance, the implicit spatial meaning of “ATTIC” was top whereas “BASEMENT” was bottom. In the congruent condition, “ATTIC” was presented at the top of the screen and “BASEMENT” at the bottom of the screen. In the incongruent condition, “ATTIC” was presented at the bottom of the screen and “BASEMENT” was presented at the top of the screen. Participants performed better in the congruent condition than in the incongruent condition. More importantly, this phenomenon was only observed in the left visual field, while embodied cognition holds that embodied representations originate in the right hemisphere [9], [10]. Hence, spatial information plays an important role in conceptual representation.

Temporal concepts are also grounded in spatial information. This is referred to as the time-space metaphor, which stipulates that past time is related to the left space and future time is related to the right space. Time, therefore, is like an arrow that flies from left to right [11]. Kranjec et al. [12] used prescribed prepositions to influence temporal concepts and found that past prepositions were related to left space and future prepositions were related to right space. In a repetitive transcranial magnetic stimulation (rTMS) study, when rTMS was applied to the cerebellum, the relationship between spatial information and temporal concepts decreased. Thus, the cerebellum might be the major neural substrate governing this phenomenon [13]. Ouellet et al. [14] adopted a spatial cue prompt paradigm to demonstrate this phenomenon and reveal its inner mechanisms. First, participants remembered a temporal word, such as “BEFORE.” Then, two boxes appeared on both sides of the screen horizontally. A white dot flashed rapidly in one of two boxes. Participants determined the box in which the dot flashed as soon as they saw it. Past temporal words improved performance in detecting the left dot and future temporal words improved performance in detecting the right dot. Moreover, the attention shifting elicited by temporal words might facilitate cause this effect. It is clear that the processing of temporal concepts relies on spatial information.

This kind of concept-space metaphor is also found in affective words, and is called the valence-space metaphor. Meier and Robinson [1] found that participants evaluated positive words faster when they were presented at the top of the screen, whereas participants evaluated negative words faster when they were presented at the bottom of the screen. Further, positive words facilitated detection of top stimuli and negative words facilitated detection of bottom stimuli. While the cognitive system is based on bodily states, the body also moderates this valence-space metaphor. Casasanto [15] proposed the body-specificity hypothesis and found that right-handers prefer to associate right space with good and left space with bad. However, left-handers prefer to associate left space with good and right space with bad. Thus, there appears to be a tendency to attribute positivity to the space more often used. de la Vega et al. [16] conducted several experiments to support the body-specificity hypothesis, and they further hold that this valence-space metaphor might not be automatic, as it required a task with explicit response mapping. Participants made a lexical judgment or a valence judgment in different experiments. The valence-by-side interaction emerged only in the valence judgment task. In addition, if the explicit mapping of valence and side was absent, the valence-by-side interaction disappeared. Although several studies have found that affective words facilitate performance on spatial task, Gozli et al. [17] discussed facilitation and interference effects in valence-space metaphors, finding that facilitation effects were more common. Interference was found when experiments used multiple concept categories and a visual discrimination task in short stimulus onset asynchrony (SOA) (200–400 ms). Gozli et al. [18], using eye-tracker technology, found that processing positive words increased the salience of the segment above fixation regardless of whether vertical distractors were present. Processing of affective words automatically recruits spatial features along vertical space. Hence, affective representation is grounded in space, and body-related variables, such as dominant hand, moderate this valence-space metaphor.

Findings regarding the concept-space metaphor come from both concrete and abstract concepts, including time, mathematical number, moral concepts, power concepts, and so forth [11], [15]–[23]. Thus, people might use spatial information to process concepts. Furthermore, embodiment was found in other animals, and these animals used perceptual information to support their behavior. For instance, rhesus monkeys demonstrated sensitivity to the time-space metaphor [24], [25].

Previous studies have demonstrated support for embodied cognition, particularly in the relationship between concepts and spatial information, such as the valence-space metaphor. The existence of this concept-space metaphor has been demonstrated across different concepts. However, the mechanism by which these valence-space metaphors affect spatial processing remains unknown. It may be that processing affective words activated spatial information and spatial information allocated attention to corresponding locations. Alternatively, processing affective words might cause people to prepare movements toward corresponding locations, because positive emotions accompany upward behaviors and negative emotions accompany downward behaviors in daily life.

Therefore, in the present study, we investigated the inner mechanisms of the valence-space metaphor. Because the valence-space metaphor has been demonstrated previously, it is permissible to test the inner mechanisms of the valence-space metaphor. Although emotional theories have not been updated as frequently as other cognitive theories, and affective word processing is a vital component of emotional theories, testing affective words might also assist in understanding emotion in affective word processing. In this experiment, a spatial cue detection paradigm was used to test how processing of affective words affects spatial cue detection. Event-related potentials (ERPs) were recorded in order to investigate the electrophysiological underpinnings of valence-space metaphor processing. ERPs have outstanding temporal resolution and significant advantages in measuring early stages of cognitive processing. Therefore, ERPs are an ideal methodology for testing the time course of the valence-space metaphor. Further, we conducted a second behavioral experiment to test the nature of attention in the valence-space metaphor. In the behavioral experiment, we used arrows as spatial cues. Arrows pointing either up or down were presented at the top or bottom of the screen. Participants were required to judge the direction in which arrows were pointing and ignore their locations on the screen. Therefore, the direction of arrows at different locations was counterbalanced, as upward facing arrows could be found in the upper and lower fields of the screen and vice versa. Consequently, we hoped to find independent effects of attention in the processing of affective words. In addition, judging arrow direction and ignoring location is an implicit method for testing attention. Participants were instructed to pay attention to the direction but not the location of the arrows.

Above all, the aim of this study was to provide electrophysiological evidence for the processing of the valance-space metaphor. Further, we conducted a behavioral experiment to test the attentional nature of the valence-space metaphor. In the ERP experiment, we hypothesized that some early ERP components might be enhanced in the metaphorical congruency condition after spatial cue presentation, because spatial cues are simple and might not result in many higher-order cognitive processes. In the behavioral experiment, we hypothesized that the metaphorical congruency effect would be found between affective word processing and arrow location, even if the task were unrelated to arrow location.

## Experiment 1

### Method

#### Ethics statement

All participants provided written informed consent prior to the experiment. They were informed of their right to withdraw at any time. The study was approved by the Human Research Ethics Committee of South China Normal University.

#### Participants

Twenty-four undergraduate students from South China Normal University, Guangzhou, China, participated in this study (mean age  = 21.50 years, *SD*  = 2.08, 19 females). We posted an advertisement on the campus forum to recruit participants and participation was voluntary. All participants were right-handed and had normal or corrected-to-normal vision. None of them had taken medicine or coffee for 24 hours before the experiment. After the experiment, participants were paid for their participation. All participants were native Chinese speakers.

#### Materials

We used 240 affective words that were selected from the Chinese Affective Words System (CAWS) [26]. We selected 120 positive words from the most positive affective words and 120 negative words from the most negative affective words. All words were two-character words, such as 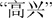 (“happy” in English). Words were matched for number of first character strokes (*M*_positive_ = 8.470, *M*_negative_ = 8.920), number of second character strokes (*M*_positive_ = 8.900, *M*_negative_ = 8.910), number of word strokes (*M*_positive_ = 17.370, *M*_negative_ = 17.830), and word occurrence frequency (*M*_positive_ = 50.340, *M*_negative_ = 33.230), all of which were statistically equivalent between positive and negative words (*p*s>.05). We used E-Prime (Psychological Software Tools, Inc., Sharpsburg, Pennsylvania) to present the words and record behavioral responses.

#### Electrophysiological Recording

The electroencephalogram (EEG) was recorded from 32 Ag/AgCl electrodes placed according to the International 10–20 system. EEG was recorded with NeuroScan NuAmps (Charlotte, NC, USA). All electrodes used the left mastoid as the on-line reference and re-referenced to average mastoids in off-line analysis. The forehead was used as the ground. Electrode impedances were maintained and impedances of all electrodes were below 5 kΩ. The electrical band-pass was from 0.1 to 100 Hz and stored with a digitization rate of 1000 Hz. Eye movements were recorded with four electrodes. Two unipolar-recording electrodes were fixed at the right and left external canthi to record horizontal electrooculograms (HEOG). Two other unipolar-recording electrodes, placed above and below the left eye, were used to record vertical electrooculograms (VEOG).

#### Research Design and Procedure

The study employed a 2 (valence of affective word: positive vs. negative) × 2 (location of spatial cue: top vs. bottom) within-subjects design.

The procedure was modified from that of a previous study [14]. All stimuli were presented on a black background. After completing 16 training trials, the experiment began. Each trial started with a centrally presented white fixation shown for 500 ms. Then, a white affective word was showed in the center of the screen (bold, 24-point SimSun Font) for 1500 ms. Participants were instructed to remember this word for the following test. After a 500 ms blank screen, two empty square boxes (1.3 cm×1.3 cm) were presented at the top and bottom of the screen for 250 ms. When the two boxes disappeared, a blank screen was presented for 500 ms. Then, a 5 mm white dot was presented in one of the two boxes for 500 ms. Participants were asked to remember where the dot was presented (no behavioral action was needed) as soon as they detected the dot. After a 500 ms blank screen, the two boxes again appeared on the screen for 1500 ms and participants were asked to respond by pressing the corresponding key. If the white dot was presented at the top, participants were instructed to press the “Y” key. If the white dot was presented at the bottom, they were instructed to press the “B” key. After a 500 ms blank screen, a screen with a question 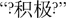 (“?Positive?”) or 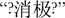 (“?Negative?”) was presented for 2500 ms or until participants responded. Participants were instructed to judge whether the previous affective word matched the question and press “Y” or “B” key for “Yes” or “No,” respectively. The response key set was counterbalanced among participants. Half pressed “Y” for “Yes,” and the other half pressed “B” for “Yes.”

Before the experiment, participants washed and blow-dried their hair in the laboratory. During the experiment, participants were asked to remain still and blink as little as possible because movements influence the EEG.

The experiment was divided into three blocks. Participants had a one-minute rest interval between the two blocks. After one minute, they were asked whether they wanted to continue to rest or begin the experiment.

### Results

#### Behavioral Results

Erroneous trials (335 trials, 5.816%) in the location detection task and the memory task were first discarded as outliers. Correct trials with reaction times below 100 ms, above 1000 ms (229 trials, 3.976%), or beyond the ±3 SD range (66 trials, 1.146%) were discarded for the location detection task; correct trials with reaction times below 300 ms, above 3000 ms (0 trials), or beyond the ±3 SD range (56 trials,.972%) were discarded for the memory task.

Reaction time and accuracy data from these two tasks were submitted to a 2 (valence of affective word: positive vs. negative) × 2 (location of spatial cue: top vs. bottom) repeated measures ANOVA or a 2 (valence of affective word: positive vs. negative) × 2 (location of spatial cue: top vs. bottom) × 2 (valence of test word: positive vs. negative) × 2 (response key set: up-yes vs. up-no) mixed ANOVA for the location detection task and memory task, separately (see Tables 1 and 2). Response key set was the key set used in the memory task, which included two types (up-yes and up-no).

**Table 1:** Mean reaction times (ms), accuracy (ACC), and standard errors (*SE*) for location detection task in Experiment 1.

	Memory Words							
	Negative				Positive			
	RT		ACC		RT		ACC	
Locations	*M*	*SE*	*M*	*SE*	*M*	*SE*	*M*	*SE*
Bottom	381.383	24.786	.994	.004	386.991	25.257	.992	.003
Top	389.761	25.363	.991	.006	392.624	24.410	.994	.003

**Table 2:** Mean reaction times (ms), accuracy (ACC), and standard errors (*SE*) for memory task in Experiment 1.

			Memory Words							
			Negative				Positive			
			RT		ACC		RT		ACC	
Response Key Set	Locations	Test Words	*M*	*SE*	*M*	*SE*	*M*	*SE*	*M*	*SE*
Up-Yes	Bottom	Negative	941.696	59.218	.906	.019	992.994	46.071	.931	.015
Up-Yes	Bottom	Positive	926.657	47.171	.975	.009	725.557	35.155	.958	.011
Up-Yes	Top	Negative	999.947	68.057	.922	.026	931.616	49.190	.953	.016
Up-Yes	Top	Positive	893.588	51.777	.969	.010	732.145	43.150	.950	.013
Up-No	Bottom	Negative	980.631	59.218	.906	.019	909.773	46.071	.972	.015
Up-No	Bottom	Positive	933.030	47.171	.975	.009	769.922	35.155	.967	.011
Up-No	Top	Negative	1029.002	68.057	.889	.026	951.877	49.190	.953	.016
Up-No	Top	Positive	981.734	51.777	.969	.010	780.006	43.150	.975	.013

**Figure 1:**
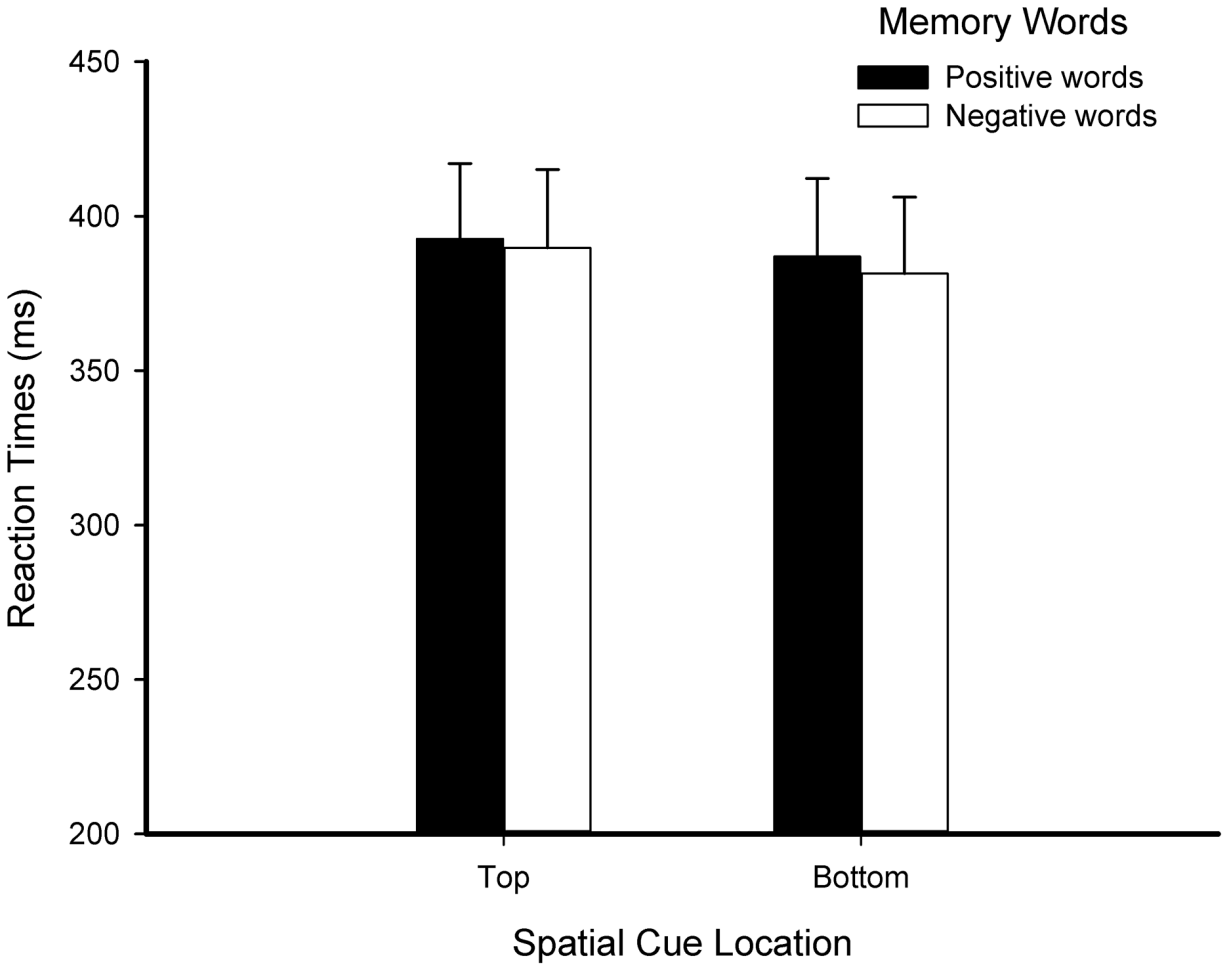
Reaction times for location detection task in Experiment 1.

In the location detection task, there were no significant effects for reaction time, *p*s>.05. In the accuracy analysis, only the interaction between memory word and location of the spatial cue was marginally significant, *F*(1, 23)  = 4.000, *p* = .057, *η_p_* ^2^ = .148. Simple effects analysis found that when spatial cues were presented at the top of the screen, there was no difference in accuracy, *F*(1, 23)  = .857, *p* = .364, *η_p_* ^2^ = .036 (*M*_positive_ = .994, *M*_negative_ = .991). A similar effect was found when spatial cues were presented at the bottom of the screen, *F*(1, 23)  = .683, *p* = .417, *η_p_* ^2^ = .029 (*M*_positive_ = .992, *M*_negative_ = .994) (see Figure 1).

In the memory task analysis, valence of affective word, location of spatial cue, and valence of test word were within-subjects factors, while response key set was a between-subjects factor. In the reaction time analysis, we found that the main effect for memory word was significant, *F*(1, 22)  = 71.440, *p* <. 0005, *η_p_* ^2^ = .765 (*M*_positive_ = 849.236 ms, *M*_negative_ = 960.786 ms). The main effect for spatial cue location was not significant, *F*(1, 22)  = 3.016, *p* = .096, *η_p_* ^2^ = .121 (*M*_top_ = 912.489 ms, *M*_bottom_ = 897.533 ms). The main effect for test word was significant, *F*(1, 22)  = 48.450, *p*<.0005, *η_p_* ^2^ = .688 (*M*_positive_ = 842.830 ms, *M*_negative_ = 967.192 ms). The main effect for response key set was not significant, *F*(1, 22)  = .137, *p* = .715, *η_p_* ^2^ = .006 (*M*_up-yes_ = 893.025 ms, *M*_up-no_ = 916.997 ms). The interaction between spatial cue location and response key set was significant, *F*(1, 22)  = 6.740, *p* = .016, *η_p_* ^2^ = .235. The interaction between memory word and test word was significant, *F*(1, 22) = 29.957, *p*<.0005, *η_p_* ^2^ = .577. These two interactions were also moderated by a 4-way interaction. The 4-way interaction for memory word, spatial cue location, test word, and response key set was significant, *F*(1, 22) = 6.393, *p* = .019, *η_p_* ^2^ = .225. No further effects reached statistical significance, *p*s>.05.

In order to interpret this 4-way interaction, we conducted two separate 3-way ANOVAs according to the response key set. When the response key set was up-yes, we found a significant main effect for memory word, *F*(1, 11) = 30.927, *p*<.0005, *η_p_* ^2^ = .738 (*M*_positive_ = 845.578 ms, *M*_negative_ = 940.472 ms). The main effect for spatial cue location was not significant, *F*(1, 11) = .646, *p* = .438, *η_p_* ^2^ = .055 (*M*_top_ = 889.324 ms, *M*_bottom_ = 896.726 ms). The main effect for test word was significant, *F*(1, 11) = 29.514, *p*<.0005, *η_p_* ^2^ = .728 (*M*_positive_ = 819.487 ms, *M*_negative_ = 966.563 ms). The interaction between memory word and test word was significant, *F*(1, 11) = 19.069, *p* = .001, *η_p_* ^2^ = .634. This 2-way interaction was also moderated by a 3-way interaction between memory word, spatial cue location, and test word, *F*(1, 11) = 8.177, *p* = .016, *η_p_* ^2^ = .426. Simple effects analysis for the 3-way interaction revealed that when test words were positive, spatial cues were top located, and when memory words were positive, participants had faster reaction times than when memory words were negative, *F*(1, 11) = 59.763, *p*<.0005, *η_p_* ^2^ = .845 (*M*_positive_ = 732.145 ms, *M*_negative_ = 893.588 ms). When test words were positive, spatial cues were bottom located, and when memory words were positive, participants had faster reaction times than when memory words were negative, *F*(1, 11) = 78.137, *p*<.0005, *η_p_* ^2^ = .877 (*M*_positive_ = 725.577 ms, *M*_negative_ = 926.657 ms). However, no such effects were found when test words were negative, regardless of whether spatial cues were located at the top, *F*(1, 11) = 2.180, *p = *.168, *η_p_* ^2^ = .165 (*M*_positive_ = 931.616 ms, *M*_negative_ = 999.947 ms), or bottom of the screen, *F*(1, 11) = 2.311, *p = *.157, *η_p_* ^2^ = .174 (*M*_positive_ = 992.994 ms, *M*_negative_ = 941.696 ms).

When the response key set was up-no, the main effects for memory word, spatial cue location, and test word were all significant, *F*(1, 11) = 40.529, *p*<.0005, *η_p_* ^2^ = .787, *F*(1, 11) = 6.571, *p = *.026, *η_p_* ^2^ = .374, and *F*(1, 11) = 18.996, *p = *.001, *η_p_* ^2^ = .633, respectively. When memory and test words were positive, participants responded to the memory task faster than when memory and test words were negative (*M*_positive_ = 852.895 ms, *M*_negative_ = 981.100 ms for memory words; *M*_positive_ = 866.173 ms, *M*_negative_ = 967.821 ms for test words). When the spatial cue was presented at the bottom of the screen, participants responded faster in the memory task (*M*_top_ = 935.655 ms, *M*_bottom_ = 898.339 ms). The interaction between memory word and test word was also significant, *F*(1, 11) = 10.945, *p = *.007, *η_p_* ^2^ = .499. Further simple effects analysis found that when memory words were positive, participants had faster reaction times than when memory words were negative, regardless of whether test words were positive, *F*(1, 11) = 46.646, *p*<.0005, *η_p_* ^2^ = .809 (*M*_positive_ = 774.964 ms, *M*_negative_ = 957.382 ms), or negative, *F*(1, 11) = 8.625, *p = *.014, *η_p_* ^2^ = .439 (*M*_positive_ = 930.825 ms, *M*_negative_ = 1004.817 ms). No further effects reached statistical significance in these two 3-way ANOVAs, *p*s>.05.

In the accuracy analysis, we found two significant main effects for memory word and test word, *F*(1, 22) = 5.689, *p = *.026, *η_p_* ^2^ = .205 and *F*(1, 22) = 23.323, *p*<.0005, *η_p_* ^2^ = .515, respectively. When memory words or test words were positive, participants had higher accuracy in the memory task (*M*_positive_ = .957, *M*_negative_ = .939 for memory words; *M*_positive_ = .967, *M*_negative_ = .929 for test words). The main effect for spatial cue location was not significant, *F*(1, 22) = .029, *p = *.866, *η_p_* ^2^ = .001 (*M*_top_ = .948, *M*_bottom_ = .949). The main effect for response key set was also not significant, *F*(1, 22) = .158, *p = *.695, *η_p_* ^2^ = .007 (*M*_up-yes_ = .945, *M*_up-no_ = .951). The interaction between memory word and test word was significant, *F*(1, 22) = 14.660, *p = *.001, *η_p_* ^2^ = .400. No other effects reached statistical significance in this ANOVA, *p*s>.05.

As we did not find significant main effect or interactions for response key set in the accuracy analysis, we discarded this between-subjects factor and conducted a 3-way repeated measures ANOVA in order to investigate the significant interaction between test word and memory word. The main effect for memory word was significant, *F*(1, 23) = 5.217, *p = *.032, *η_p_* ^2^ = .185 (*M*_positive_ = .957, *M*_negative_ = .939). The main effect for spatial cue location was not significant, *F*(1, 23) = .029, *p = *.867, *η_p_* ^2^ = .001 (*M*_top_ = .948, *M*_bottom_ = .949). The main effect for test word was significant, *F*(1, 23) = 24.215, *p*<.0005, *η_p_* ^2^ = .513 (*M*_positive_ = .967, *M*_negative_ = .929). The interaction between memory word and test word was significant, *F*(1, 23) = 14.984, *p = *.001, *η_p_* ^2^ = .394. Follow-up analyses revealed that when the test word was positive, memory words did not affect performance on the memory task, *F*(1, 23) = 1.119, *p = *.301, *η_p_* ^2^ = .046 (*M*_positive_ = .962, *M*_negative_ = .972). When the test word was negative, positive memory words enhanced accuracy on the memory test, *F*(1, 23) = 14.342, *p = *.001, *η_p_* ^2^ = .384 (*M*_positive_ = .952, *M*_negative_ = .906). No further effects reached statistical significance, *p*s>.05.

#### ERP Results

The Neuroscan Scan 4.5 (Charlotte, NC, USA) was used for data analysis. First, in off-line analysis, the reference was changed from the left mastoid into the average of two mastoids. Then, unipolar recordings in HEOG and VEOG were transformed into bipolar recordings and eye artifacts were corrected. Erroneous trials in the memory task were first discarded as outliers. Recording channel artifacts were rejected by looking at whether the time domain was outside of the −80 µV to 80 µV range. Two subjects were excluded due to excess artifacts. Stimulus-locked ERPs were analyzed with a −100 ms baseline to 800 ms post-stimulus. The off-line band-pass filter was set from 0 Hz to 30 Hz, 24 dB/oct.

**Figure 2:**
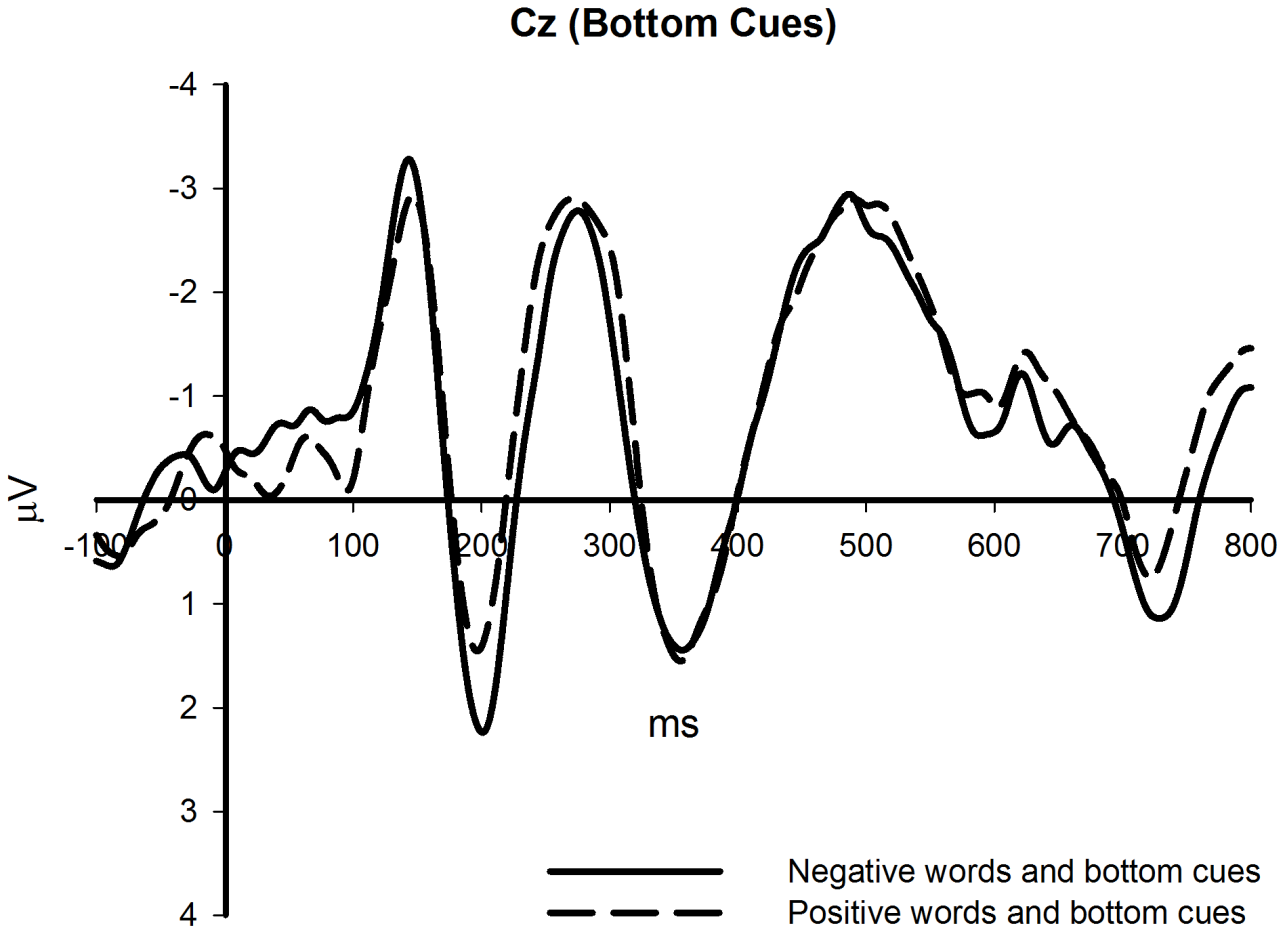
The effects of different affective words on bottom cues (Cz site).

**Figure 3:**
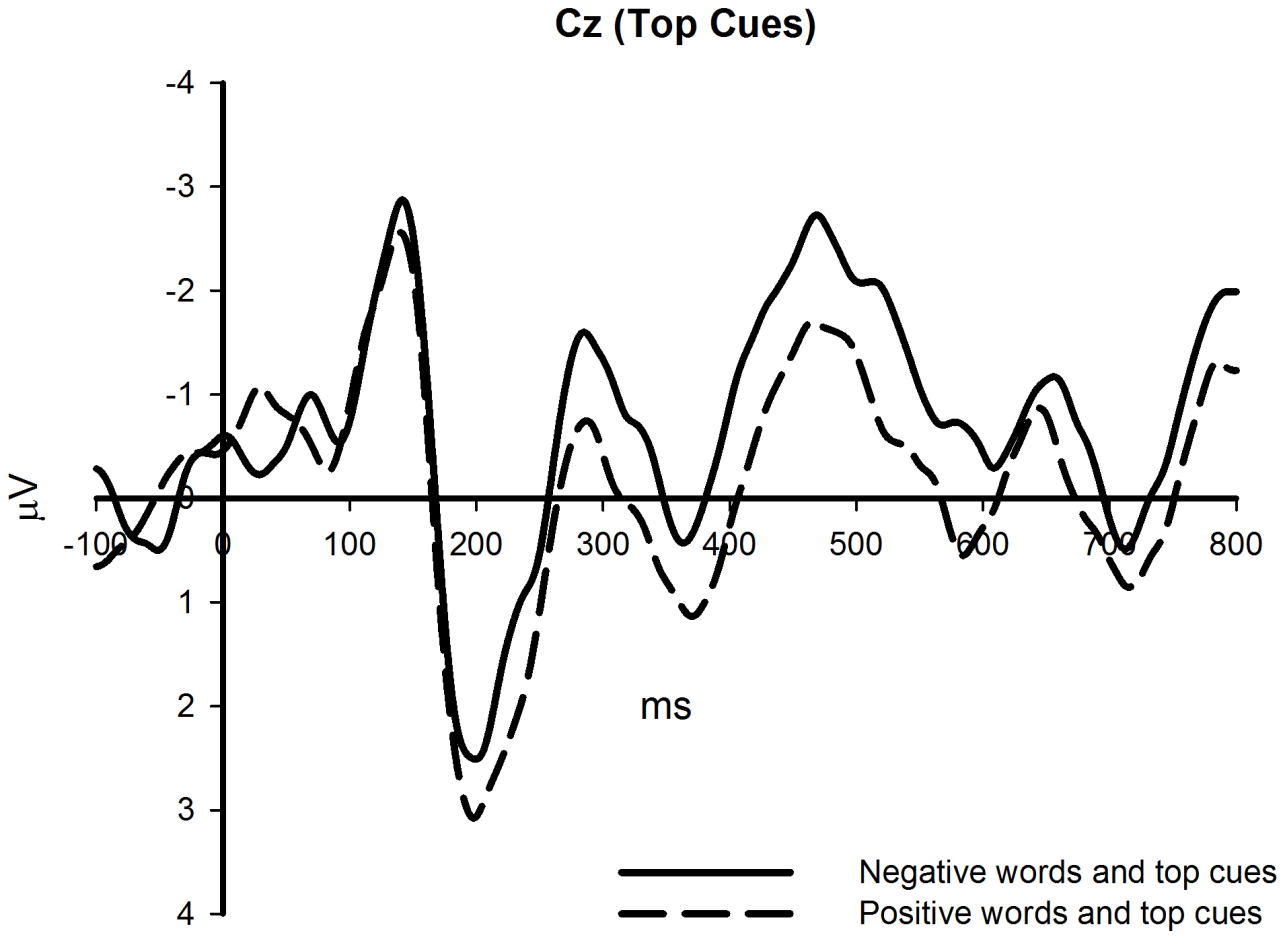
The effects of different affective words on top cues (Cz site).

**Figure 4:**
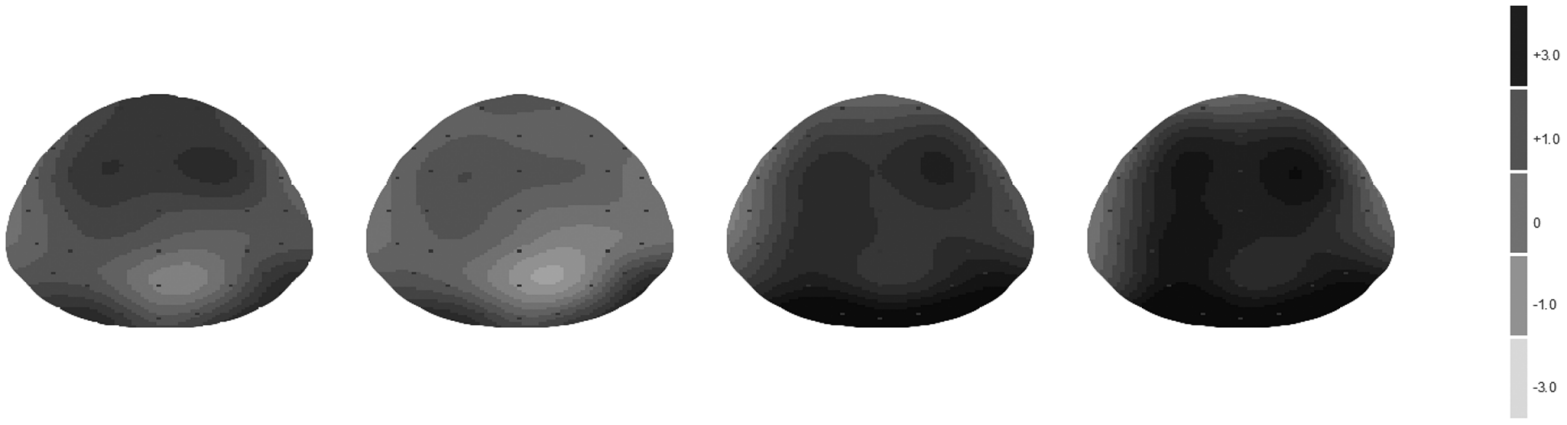
Voltage scalp maps of the P200 for four different trial types.

Significant differences were revealed for mean amplitude around 200 ms after the presentation of spatial cues. This was a positive component with a latency around 200 ms post-stimulus, and thus, was likely the P200. There was a larger P200 potential around 200 ms for top cues after positive words and for bottom cues after negative words. Consistent with previous studies, the P200 was quantified by a mean amplitude measure for F3, Fz, F4, FC3, FCz, FC4, C3, Cz, and C4, which were the front and middle electrodes [27], [28] (see Figures 2–4).

Mean amplitude measures between 190 ms and 240 ms were selected for further analysis [27], [28]. Mean amplitude measures were then calculated for each of the 36 trial types for each subject, and were submitted to a 2 (valence of affective word: positive vs. negative) × 2 (location of spatial cue: top vs. bottom) × 9 (electrode: F3, Fz, F4, FC3, FCz, FC4, C3, Cz, and C4) repeated measures ANOVA. The results were adjusted using the Greenhouse-Geisser method.

**Figure 5:**
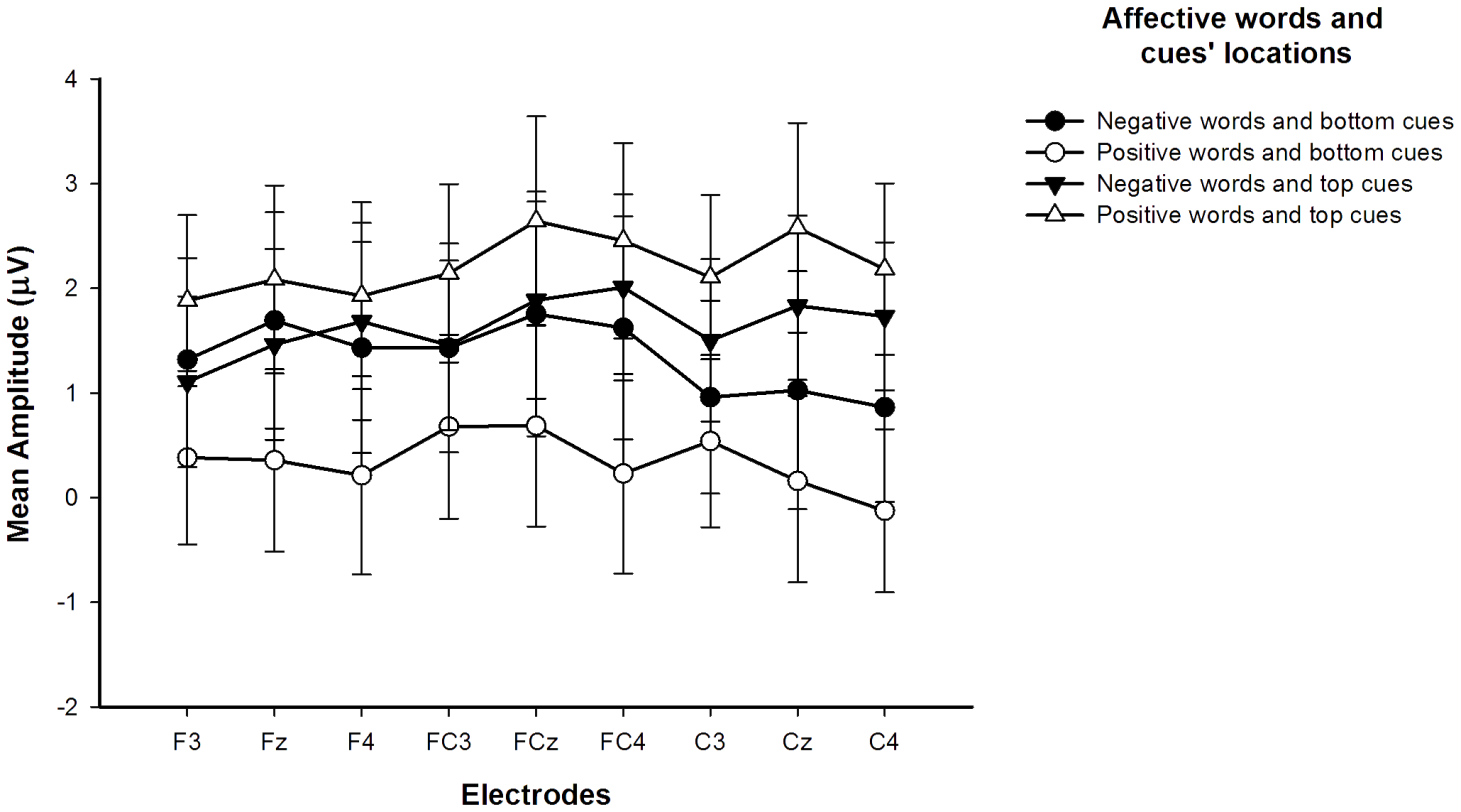
Mean amplitude for four different trial types at nine electrode sites.

The ANOVA for mean amplitude measures revealed that the main effect for valence of memory word was not significant, *F*(1, 21) = .622, *p = *.439, *η_p_* ^2^ = .029 (*M*_positive_ = 1.285 µV, *M*_negative_ = 1.488 µV). The main effect for the spatial cue location was significant, *F*(1, 21) = 11.470, *p = *.003, *η_p_* ^2^ = .353. Further analysis revealed that top spatial cues elicited larger potentials around 200 ms post-stimulus than did bottom spatial cues (*M*_top_ = 1.928 µV, *M*_bottom_ = .846 µV). The main effect for electrode was not significant, *F*(8, 168) = .821, *p = *.502, *η_p_* ^2^ = .038. Most importantly, the interaction between valence of memory word and the location of the spatial cue was significant, *F*(1, 21) = 6.631, *p = *.018, *η_p_* ^2^ = .240. Simple effects analysis revealed that when the spatial cues were presented at the bottom, negative words induced a larger P200 than did positive words, *F*(1, 21) = 5.901, *p = *.024, *η_p_* ^2^ = .219 (*M*_positive_ = .346 µV, *M*_negative_ = 1.346 µV). When the spatial cues appeared in the top location, there was no difference in P200 amplitude between positive and negative words, *F*(1, 21) = 2.272, *p = *.147, *η_p_* ^2^ = .098 (*M*_positive_ = 2.224 µV, *M*_negative_ = 1.631 µV). Meanwhile, when participants kept positive words in mind, top spatial cues elicited larger P200 amplitudes than did bottom spatial cues, *F*(1, 21) = 30.866, *p*<.0005, *η_p_* ^2^ = .595 (*M*_top_ = 2.224 µV, *M*_bottom_ = .346 µV). When participants kept negative words in mind, there was no difference in P200 amplitude between top and bottom spatial cues, *F*(1, 21) = .290, *p = *.596, *η_p_* ^2^ = .014 (*M*_top_ = 1.631 µV, *M*_bottom_ = 1.346 µV). The interaction between valence of memory word and electrode was significant, *F*(8, 168) = 3.150, *p = *.021, *η_p_* ^2^ = .130. Simple effects analysis found that different types of memory words did not affect P200 amplitude at the nine sites, *p*s>.05. The interaction between spatial cue location and electrode was significant, *F*(8, 168) = 8.589, *p*<.0005, *η_p_* ^2^ = .290. Simple effects analysis revealed that spatial cue locations showed significant differences at the F4, C3, Cz, C4, FC3, FCz, and FC4 sites, *p*s<.05. Spatial cue locations showed marginally significant differences at the F3 and Fz sites, *p*s<.10. Hence, when the spatial cues were presented at the top, they induced more pronounced P200s than did bottom spatial cues at all sites. The 3-way interaction between valence of memory word, spatial cue location, and electrode was not significant, *F*(8, 168) = .991, *p = *.402, *η_p_* ^2^ = .045 (see Table 3 and Figure 5).

**Table 3:** Mean amplitudes (μV) and standard errors (*SE*) in four different trial types.

	Negative Words		Positive Words	
	*M*	*SE*	*M*	*SE*
Bottom Spatial Cues	1.346	1.005	.346	.867
Top Spatial Cues	1.631	.825	2.224	.861

### Discussion

In the location detection task, we did not find any significant effects for reaction time, likely because this location detection task was offline, and participants had to wait for 1000 ms from the presentation of target stimuli to respond. Meanwhile, we found only a marginally significant interaction between memory word and location of spatial cue in accuracy. Although simple effects analysis did not reveal any significant findings, there was a tendency towards a metaphorical congruency effect. When spatial cues were presented at the top or bottom of the screen, participants had higher accuracy when they kept positive or negative words in mind, respectively. This tendency is consistent with our hypothesis that positive words facilitate the processing of top spatial cues, while negative words facilitate the processing of bottom spatial cues.

In the memory task, we found a significant 4-way interaction for memory word, spatial cue location, test word, and response key set on reaction time. We then conducted two separate ANOVAs to interpret this interaction. When response key set was up-yes, we found that when test words and memory words were positive, participants had faster reaction times than when memory words were negative. This finding indicates that positive memory words facilitate participants’ recall when test words are also positive. However, the absence of this effect for negative test words might be because participants are more sensitive to positive words. For example, we found that participants responded to positive memory and test words faster than for negative memory and test words. Further, when the response key set was up-no, we also found that participants had faster reaction times when memory words were positive. Hence, positive words had a greater influence on the memory task, regardless of response key set. We will conduct an additional behavioral experiment to determine whether this effect is stable, given that the current behavioral data were offline. For accuracy, we found that positive memory words facilitated participants’ responses in the memory task. However, this effect was only significant when test words were negative. The accuracy data also indicated that positive words had a greater influence on the memory test. We will conduct another behavioral experiment to test whether this effect was caused by asymmetric processing for valence words.

In the ERP results, we found that memory words affected performance on the spatial cue location task. When memory spatial cues were presented at the top or bottom of the screen, the amplitude of the P200 was larger when participants kept positive or negative words in mind, respectively. This effect was only significant when spatial cues appeared at the bottom of the screen. Meanwhile, when participants kept positive words in mind, top spatial cues induced larger P200 amplitudes. However, such an effect was not found when participants kept negative words in mind. Hence, we found a metaphorical congruency effect in the ERP results, which supported our hypothesis. The effect of affective words on the spatial cue detection task could be explained in several ways. First, valence words activated attention allocation and facilitated performance on the spatial detection task. Second, valence words primed response preparation, and this preparation elicited larger P200 amplitudes. We will discuss these potential explanations in the general discussion section. In addition, we also found that when spatial cues were presented at the top of the screen, they induced larger P200 amplitudes, which might be caused by the physical features of spatial cues, and thus might be unrelated to the metaphorical congruency effect.

In summary, affective words affected mean P200 amplitude as a function of spatial cue. Bottom spatial cues appearing immediately after negative words induced a larger P200 than after positive words; top spatial cues induced a larger P200 when presented immediately after positive words. These findings might be related to attention allocation because of the early latency of the P200. However, the behavioral results did not reveal any significant findings that related to our main hypothesis. This might be because participants were required to respond only after keeping the location of the spatial cue in mind. Therefore, the test was an offline test that does not reflect participants’ online behavioral responses. Further, the main finding that affective words affected P200 amplitude as a function of spatial cue in Experiment 1 might also be explained by response preparation. We planned to acquire online behavioral data and dissociate response preparation from attention allocation in the following experiment.

Hence, we conducted Experiment 2 using behavioral methods to test online behavioral responses and dissociate response preparation from attention allocation. In Experiment 2, we also considered attention allocation and response preparation. The dot was replaced by arrows pointing up or down, because we wanted to separate attention allocation from response preparation [14]. If we continued to use the dot as the target cue, and changed its features to fit the following experiment, we might have induced other metaphors, such as valence-color or valence-shape metaphors. By using arrows as target cues, we could distinguish the differential contributions of arrow location and direction to the valence-space metaphor, as arrow location and direction are only related with space [29], [30]. In Experiment 2, participants judged the direction of arrows, ignoring their location. In this way, arrow location was invalid information for completing the experimental task. Therefore, regardless of arrow location, participants had to press both upper and lower keys to respond. However, arrow location might be the primary attractor of participants’ attention, and thus result in attention allocation. Arrow direction might induce participants’ response preparation. Because we want to distinguish response preparation from attention allocation, arrow location and arrow direction were used in Experiment 2. If attention allocation independently produced the valence-space metaphor, an interaction between affective word and arrow location would be revealed; if response preparation also participated in this metaphor alone or concomitantly, a 2-way interaction between affective word and arrow direction, or a 3-way interaction between affective word, arrow location, and arrow direction would be observed.

## Experiment 2

### Method

#### Ethics statement

All participants provided written informed consent prior to the experiment. They were informed of their right to withdraw at any time. The study was approved by the Human Research Ethics Committee of South China Normal University.

#### Participants

Thirty-two undergraduate students from South China Normal University, Guangzhou, China, participated in this study for monetary compensation (mean age = 20.25 years, *SD* = 1.81, 29 females). None participated in the previous experiment. They were randomly recruited through an advertisement posted on the campus forum. All were right-handed and had normal or corrected-to-normal vision.

#### Materials

We selected 192 affective words (96 positive words and 96 negative words) from the CAWS [26]. Word lists were matched for number of first character strokes (*M*_positive_ = 8.540, *M*_negative_ = 8.830), number of second character strokes (*M*_positive_ = 8.750, *M*_negative_ = 8.570), number of word strokes (*M*_positive_ = 17.292, *M*_negative_ = 17.406), and word occurrence frequency (*M*_positive_ = 43.020, *M*_negative_ = 40.060), all of which revealed non-significant differences (*p*s>.05). We used E-Prime (Psychological Software Tools, Inc., Sharpsburg, Pennsylvania) to present the experimental materials and record behavioral responses.

#### Research Design and Procedure

The study was a 2 (valence of affective word: positive vs. negative) × 2 (location of arrow: top vs. bottom) × 2 (direction of arrow: up vs. down) within-subjects design.

The procedure was similar to that used in Experiment 1, but the two empty squares were removed and the dot was replaced by white arrows (.69 cm×.74 cm) pointing up or down. Affective words were presented in the center of the screen (bold, 30-point SimSun Font) for 1500 ms before presentation of arrow. After a 500 ms blank screen, arrows were presented at the bottom or top of the screen for 50 ms. Participants were instructed to indicate as soon as possible whether the arrow pointed up or down, ignoring arrow location. Participants then recalled affective words and responded.

### Results

Two participants were removed due to low accuracy in the memory recognition task (lower than 80%). Incorrect trials (553 trials, 9.60%) in the direction discrimination task and the memory task were first discarded as outliers. Correct trials with reaction times below 200 ms, above 1500 ms (37 trials,.642%), or outside of the ±3 SD range (56 trials,.972%) were discarded for the arrow detection task; correct trials with reaction times below 300 ms, above 3000 ms (117 trials, 2.031%), or outside of the ±3 SD range (2 trials,.035%) were discarded for the memory recognition task.

Reaction time and accuracy data from these two tasks were submitted to a 2 (valence of affective word: positive vs. negative) × 2 (location of arrow: top vs. bottom) × 2 (direction of arrow: up vs. down) repeated measures ANOVA or a 2 (valence of affective word: positive vs. negative) × 2 (location of arrows: top vs. bottom) × 2 (direction of arrow: up vs. down) × 2 (valence of test word: positive vs. negative) × 2 (response key set: up-yes vs. up-no) mixed ANOVA for the direction discrimination task and memory task, respectively (see Tables 4 and 5)

**Table 4:** Mean reaction times (ms), accuracy (ACC), and standard errors (*SE*) for direction discrimination task in Experiment 2.

		Memory Words							
		Negative				Positive			
		RT		ACC		RT		ACC	
Locations	Direction	*M*	*SE*	*M*	*SE*	*M*	*SE*	*M*	*SE*
Bottom	Down	508.650	12.844	.997	.002	514.447	14.239	.994	.003
Bottom	Up	586.356	17.413	.953	.012	595.082	21.058	.940	.011
Top	Down	549.992	17.021	.971	.006	546.999	15.146	.964	.014
Top	Up	531.066	15.824	.979	.006	519.926	15.606	.986	.005

**Table 5:** Mean reaction times (ms), accuracy (ACC), and standard errors (*SE*) for memory task in Experiment 2.

				Memory Words							
				Negative				Positive			
				RT		ACC		RT		ACC	
Response Key Set	Locations	Direction	Test Words	*M*	*SE*	*M*	*SE*	*M*	*SE*	*M*	*SE*
Up-Yes	Bottom	Down	Negative	1140.009	83.508	.894	.027	1273.031	88.612	.867	.021
Up-Yes	Bottom	Down	Positive	1048.267	80.284	.950	.021	887.733	75.368	.967	.016
Up-Yes	Bottom	Up	Negative	1166.826	90.588	.856	.025	1037.857	71.376	.939	.019
Up-Yes	Bottom	Up	Positive	984.733	78.810	.967	.017	857.125	76.556	.983	.013
Up-Yes	Top	Down	Negative	1142.514	84.919	.917	.030	1130.458	79.640	.900	.019
Up-Yes	Top	Down	Positive	1115.345	82.412	.906	.020	968.557	83.321	.961	.020
Up-Yes	Top	Up	Negative	1078.638	84.613	.844	.035	1010.844	86.354	.967	.027
Up-Yes	Top	Up	Positive	997.070	68.314	.956	.019	859.041	62.698	.972	.014
Up-No	Bottom	Down	Negative	1263.471	83.508	.889	.027	1327.540	88.612	.950	.021
Up-No	Bottom	Down	Positive	1238.020	80.284	.961	.021	1133.185	75.368	.944	.016
Up-No	Bottom	Up	Negative	1360.055	90.588	.872	.025	1358.340	71.376	.939	.019
Up-No	Bottom	Up	Positive	1268.318	78.810	.917	.017	1144.732	76.556	.950	.013
Up-No	Top	Down	Negative	1327.338	84.919	.872	.030	1311.598	79.640	.967	.019
Up-No	Top	Down	Positive	1269.222	82.412	.967	.020	1134.793	83.321	.944	.020
Up-No	Top	Up	Negative	1330.192	84.613	.889	.035	1353.938	86.354	.894	.027
Up-No	Top	Up	Positive	1276.521	68.314	.917	.019	1136.126	62.698	.961	.014

**Figure 6:**
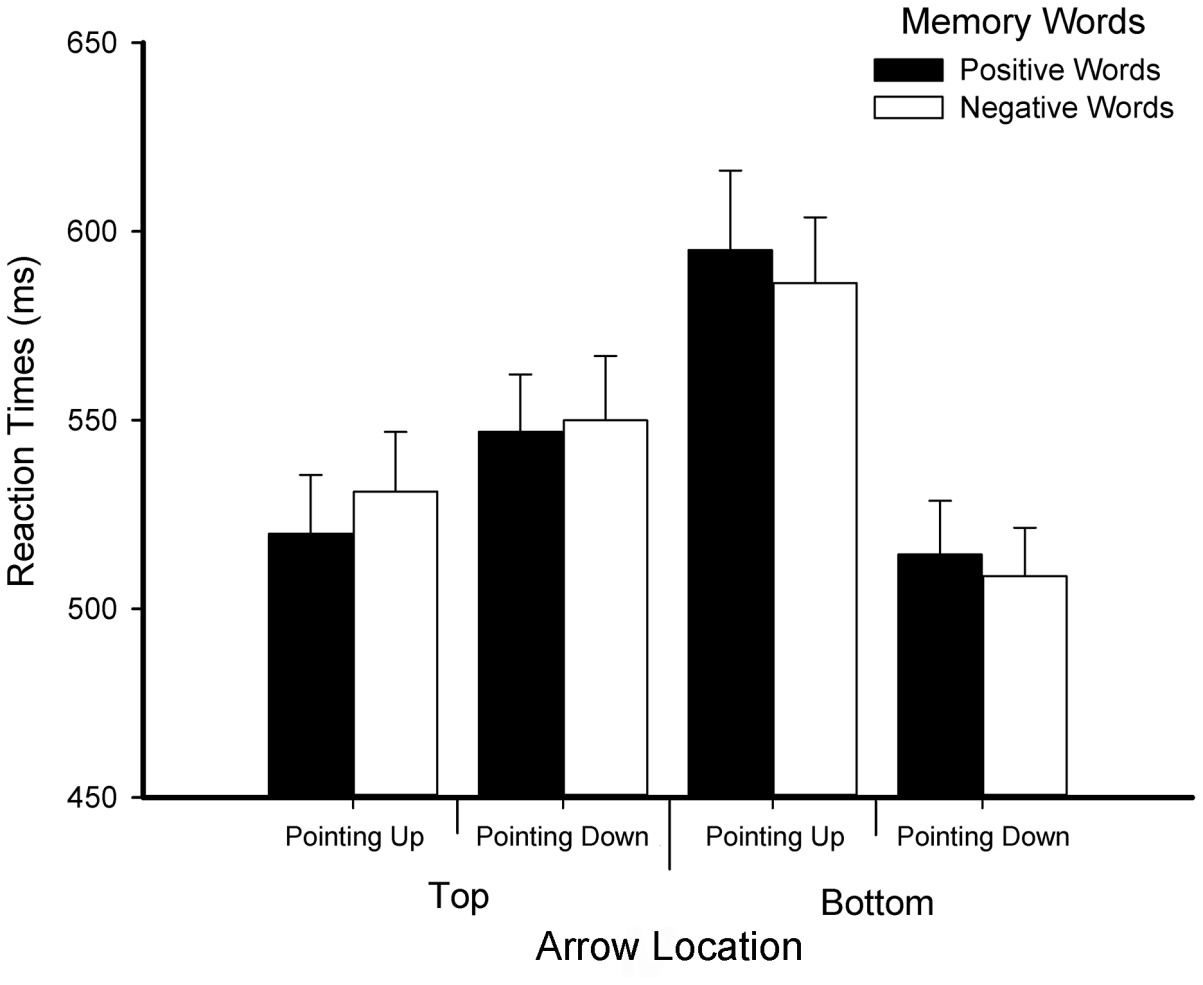
Reaction times for direction discrimination task in Experiment 2.

In the direction discrimination task, reaction time revealed significant main effects for arrow location and arrow direction, *F*(1, 29) = 10.015, *p = *.004, *η_p_* ^2^ = .257 and *F*(1, 29) = 8.417, *p = *.007, *η_p_* ^2^ = .225, respectively (*M*_top_ = 536.996 ms, *M*_bottom_ = 551.134 ms for arrow location; *M*_up_ = 558.107 ms, *M*_down_ = 530.022 ms for arrow direction). The interaction between arrow location and arrow direction was significant, *F*(1, 29) = 64.045, *p*<.0005, *η_p_* ^2^ = .688. Simple effects analysis revealed that when arrows pointed up, participants performed better for arrows that were presented at the top of the screen, *F*(1, 29) = 75.258, *p*<.0005, *η_p_* ^2^ = .722 (*M*_top_ = 525.496 ms, *M*_bottom_ = 590.719 ms). When arrows pointed down, participants performed better for arrows that were presented at the bottom of the screen, *F*(1, 29) = 21.039, *p*<.0005, *η_p_* ^2^ = .420 (*M*_top_ = 548.496 ms, *M*_bottom_ = 511.548 ms). Most importantly, the interaction between affective word and arrow location was significant, *F*(1, 29) = 6.215, *p = *.019, *η_p_* ^2^ = .176. Simple effects analysis demonstrated that when participants kept positive words in mind, response times for top arrows were faster than for bottom arrows, *F*(1, 29) = 13.016, *p = *.001, *η_p_* ^2^ = .310 (*M*_top_ = 533.463 ms, *M*_bottom_ = 554.764 ms). No similar effects were found while participants kept negative words in mind, *F*(1, 29) = 2.254, *p = *.144, *η_p_* ^2^ = .072 (*M*_top_ = 540.529 ms, *M*_bottom_ = 547.503 ms). No other significant effects were revealed, *ps*>.05 (see Figure 6).

Accuracy data revealed a significant main effect for arrow direction, *F*(1, 29) = 9.103, *p = *.005, *η_p_* ^2^ = .239. Participants were more accurate for arrows pointing down (*M*_up_ = .965, *M*_down_ = .982). The interaction between arrow location and arrow direction was significant, *F*(1, 29) = 18.387, *p*<.0005, *η_p_* ^2^ = .388. Simple effects analysis revealed that when the arrows pointed up and were presented at the top of the screen, participants had a higher accuracy than for arrows that were presented at the bottom of the screen, *F*(1, 29) = 13.345, *p = *.001, *η_p_* ^2^ = .315 (*M*_top_ = .983, *M*_bottom_ = .947). When arrows pointed down and were presented at the bottom of the screen, participants had higher accuracy than for arrows that were presented at the top of the screen, *F*(1, 29) = 9.850, *p = *.004, *η_p_* ^2^ = .254 (*M*_top_ = .967, *M*_bottom_ = .996). No further significant effects were found, *p*s>.05.

In the memory task, reaction times revealed a significant main effect for memory word, *F*(1, 28) = 29.480, *p*<.0005, *η_p_* ^2^ = .513 (*M*_positive_ = 1120.306 ms, *M*_negative_ = 1187.909 ms). The main effect for arrow location was not significant, *F*(1, 28) = .100, *p = *.754, *η_p_* ^2^ = .004 (*M*_top_ = 1152.637 ms, *M*_bottom_ = 1155.578 ms). The main effect for arrow direction was significant, *F*(1, 28) = 4.647, *p = *.040, *η_p_* ^2^ = .142 (*M*_up_ = 1138.772 ms, *M*_down_ = 1169.443 ms). The main effect for test word was also significant, *F*(1, 28) = 62.061, *p*<.0005, *η_p_* ^2^ = .689 (*M*_positive_ = 1082.424 ms, *M*_negative_ = 1225.791 ms). The main effect for response key set was significant, *F*(1, 28) = 4.700, *p = *.039, *η_p_* ^2^ = .144 (*M*_up-yes_ = 1043.628 ms, *M*_up-no_ = 1264.587 ms). The interaction between arrow direction and response key set was significant, *F*(1, 28) = 16.935, *p*<.0005, *η_p_* ^2^ = .377. The interaction between memory word and test word was significant, *F*(1, 28) = 16.626, *p*<.0005, *η_p_* ^2^ = .373. The interaction between arrow location and test word was significant, *F*(1, 28) = 7.453, *p = *.011, *η_p_* ^2^ = .210. The interaction between arrow location, test word, and response key set was significant, *F*(1, 28) = 6.227, *p = *.019, *η_p_* ^2^ = .182. The interaction between memory word, arrow direction, and test word was significant, *F*(1, 28) = 7.823, *p = *.009, *η_p_* ^2^ = .218. The interaction between memory word, arrow direction, test word, and response key set was also significant, *F*(1, 28) = 7.687, *p = *.010, *η_p_* ^2^ = .215. In order to interpret these interactions, we conducted two separate 3-way ANOVAs that were separated by response key set.

When the response key set was up-yes, we found significant main effects for memory word and test word, *F*(1, 14) = 27.652, *p*<.0005, *η_p_* ^2^ = .664 and *F*(1, 14) = 41.099, *p*<.0005, *η_p_* ^2^ = .746, respectively. When memory words or test words were positive, participants had faster response times on the memory task (*M*_positive_ = 1003.081 ms, *M*_negative_ = 1084.175 ms for memory words; *M*_positive_ = 964.734 ms, *M*_negative_ = 1122.522 ms for test words). The main effect for arrow direction was significant, *F*(1, 14) = 20.766, *p*<.0005, *η_p_* ^2^ = .597 (*M*_up_ = 999.017 ms, *M*_down_ = 1088.239 ms). However, the main effect for arrow location was not significant, *F*(1, 14) = .741, *p = *.404, *η_p_* ^2^ = .050 (*M*_top_ = 1037.808 ms, *M*_bottom_ = 1049.448 ms). The interaction between arrow location and test word was significant, *F*(1, 14) = 14.669, *p = *.002, *η_p_* ^2^ = .512. Simple effects analysis revealed that when test words were negative and arrows were presented at the top of the screen, participants had faster reaction times than for arrows that were presented at the bottom of the screen, *F*(1, 14) = 11.720, *p = *.004, *η_p_* ^2^ = .456 (*M*_top_ = 1090.614 ms, *M*_bottom_ = 1154.430 ms). However, no such effect was found when test words were positive, *F*(1, 14) = 4.218, *p = *.059, *η_p_* ^2^ = .232 (*M*_top_ = 985.003 ms, *M*_bottom_ = 944.465 ms). The interaction between memory word and test word was significant, *F*(1, 14) = 8.582, *p = *.011, *η_p_* ^2^ = .380. Further, the 3-way interaction between memory word, arrow direction, and test word was significant, *F*(1, 14) = 17.945, *p = *.001, *η_p_* ^2^ = .562. As the interaction between memory word and test word was moderated by a 3-way interaction, we only conducted a simple effects analysis on the 3-way interaction. Simple effects analysis revealed that when memory and test words were positive, participants responded faster than they did for negative memory words, regardless of whether arrows pointed up, *F*(1, 14) = 16.569, *p = *.001, *η_p_* ^2^ = .542 (*M*_positive_ = 858.083 ms, *M*_negative_ = 990.902 ms), or down, *F*(1, 14) = 21.128, *p*<.0005, *η_p_* ^2^ = .601 (*M*_positive_ = 928.145 ms, *M*_negative_ = 1081.806 ms). A similar effect was found when arrows pointed up and test words were negative, *F*(1, 14) = 5.595, *p = *.033, *η_p_* ^2^ = .286 (*M*_positive_ = 1024.350 ms, *M*_negative_ = 1122.732 ms). However, such an effect was not found when arrows pointed down and test words were negative, *F*(1, 14) = 4.334, *p = *.056, *η_p_* ^2^ = .236 (*M*_positive_ = 1201.745 ms, *M*_negative_ = 1141.262 ms).

When response key set was up-no, we found significant effects for memory words and test words, *F*(1, 14) = 7.659, *p = *.015, *η_p_* ^2^ = .354 and *F*(1, 14) = 23.126, *p*<.0005, *η_p_* ^2^ = .623, respectively. When memory words or test words were positive, participants responded faster on the memory task (*M*_positive_ = 1237.531 ms, *M*_negative_ = 1291.642 ms for memory words; *M*_positive_ = 1200.115 ms, *M*_negative_ = 1329.059 ms for test words). The interaction between memory and test words was also significant, *F*(1, 14) = 8.193, *p = *.013, *η_p_* ^2^ = .369. Simple effects analysis revealed that when memory and test words were positive, participants had faster reaction times than when memory words were negative, *F*(1, 14) = 37.201, *p*<.0005, *η_p_* ^2^ = .727 (*M*_positive_ = 1137.209 ms, *M*_negative_ = 1263.020 ms). However, such an effect was not found when test words were negative, *F*(1, 14) = .194, *p = *.666, *η_p_* ^2^ = .014 (*M*_positive_ = 1337.854 ms, *M*_negative_ = 1320.264 ms). No further effects reached statistical significance in these two separate ANOVAs, *p*s>.05.

Accuracy results revealed main effects for memory word and test word, *F*(1, 28) = 11.549, *p = *.002, *η_p_* ^2^ = .292 and *F*(1, 28) = 27.830, *p*<.0005, *η_p_* ^2^ = .498, respectively (*M*_positive_ = .944, *M*_negative_ = .911 for memory words; *M*_positive_ = .951, *M*_negative_ = .903 for test words). The main effects for arrow location and arrow direction were not significant, *F*(1, 28) = .018, *p = *.894, *η_p_* ^2^ = .001 and *F*(1, 28) = .084, *p = *.774, *η_p_* ^2^ = .003, respectively (*M*_top_ = .927, *M*_bottom_ = .928 for arrow location; *M*_up_ = .926, *M*_down_ = .928 for arrow direction). The main effect for response key set was not significant, *F*(1, 28) = .002, *p = *.962, *η_p_* ^2^<.001 (*M*_up-yes_ = .928, *M*_up-no_ = .927). The interaction between arrow direction and response key set was significant, *F*(1, 28) = 5.841, *p = *.022, *η_p_* ^2^ = .173. The interaction between memory word and test word was also significant, *F*(1, 28) = 4.606, *p = *.041, *η_p_* ^2^ = .141. These two 2-way interactions were also moderated by a significant 4-way interaction between memory word, arrow direction, test word, and response key set, *F*(1, 28) = 16.527, *p*<.0005, *η_p_* ^2^ = .371. In order to interpret this 4-way interaction, we conducted two 3-way ANOVAs by response key set.

When response key set was up-yes, we found significant main effects for memory word and test word, *F*(1, 14) = 7.780, *p = *.014, *η_p_* ^2^ = .357 and *F*(1, 14) = 33.684, *p*<.0005, *η_p_* ^2^ = .706, respectively (*M*_positive_ = .944, *M*_negative_ = .911 for memory words; *M*_positive_ = .958, *M*_negative_ = .898 for test words). The interaction between arrow location and test word was significant, *F*(1, 14) = 6.789, *p = *.021, *η_p_* ^2^ = .327. Simple effects analysis revealed that when test words were negative and arrows were presented at the top of the screen, participants had higher accuracy than when arrows were presented at the bottom of the screen, *F*(1, 14) = 4.971, *p = *.043, *η_p_* ^2^ = .262 (*M*_top_ = .907, *M*_bottom_ = .889). However, this effect was not found when test words were positive, *F*(1, 14) = 2.641, *p = *.126, *η_p_* ^2^ = .159 (*M*_top_ = .949, *M*_bottom_ = .967). The interaction between memory word and arrow direction was significant, *F*(1, 14) = 4.997, *p = *.042, *η_p_* ^2^ = .263. This interaction was also moderated by a significant 3-way interaction between memory word, arrow direction, and test word, *F*(1, 14) = 14.200, *p = *.002, *η_p_* ^2^ = .504. Simple effects analysis revealed that when test words were negative, arrows pointed up, and memory words were positive, participants were more accurate in the memory task than when memory words were negative, *F*(1, 14) = 22.926, *p*<.0005, *η_p_* ^2^ = .621 (*M*_positive_ = .953, *M*_negative_ = .850). Such an effect was not found in other conditions, *p*s>.05.

When response key set was up-no, we found significant main effects for memory word, arrow direction, and test word, *F*(1, 14) = 4.591, *p = *.050, *η_p_* ^2^ = .247, *F*(1, 14) = 5.820, *p = *.030, *η_p_* ^2^ = .294, and *F*(1, 14) = 5.819, *p = *.030, *η_p_* ^2^ = .294 (*M*_positive_ = .944, *M*_negative_ = .910 for memory words; *M*_up_ = .917, *M*_down_ = .937 for arrow direction; *M*_positive_ = .945, *M*_negative_ = .909 for test words). The main effect for arrow location was not significant, *F*(1, 14) = .032, *p = *.861, *η_p_* ^2^ = .002 (*M*_top_ = .926, *M*_bottom_ = .928). The interaction between memory word and test word was significant, *F*(1, 14) = 5.164, *p = *.039, *η_p_* ^2^ = .269. Further, this interaction was moderated by a 3-way interaction between memory word, arrow direction, and test word, *F*(1, 14) = 4.659, *p = *.049, *η_p_* ^2^ = .250. Simple effects analysis found that when test words were positive, arrows pointed up, and memory words were positive, participants had higher accuracy than they did when memory words were negative, *F*(1, 14) = 5.914, *p = *.029, *η_p_* ^2^ = .297 (*M*_positive_ = .956, *M*_negative_ = .917). Meanwhile, when test words were negative, arrows pointed down, and memory words were positive, participants had higher accuracy than they did when memory words were negative, *F*(1, 14) = 7.054, *p = *.019, *η_p_* ^2^ = .335 (*M*_positive_ = .958, *M*_negative_ = .881). Such an effect was not found in other conditions, *p*s>.05. No other effects reached statistical significance in these two separate ANOVAs, *p*s>.05.

### Discussion

The behavioral experiment revealed a significant interaction between affective word and arrow location. Keeping positive words in mind facilitated the discrimination of arrows that were presented at the top of the screen. Therefore, the results demonstrated a metaphorical congruency effect when memory words were positive.

In the direction discrimination task, the interaction between arrow location and arrow direction on reaction time and accuracy seemed to be a spatial Stroop effect [31]. The relevant stimulus dimension was the arrow direction, and the irrelevant dimension was the arrow location. Participants responded more accurately when arrows were presented at congruent locations (e.g., arrows pointing up were presented at the top of the screen). Most importantly, we found that positive words facilitated participants’ responses on top arrows in reaction times, which indicated that positive words might activate attention allocation, and thus resulted in the metaphorical congruency effect. However, this finding could also be explained by other theories, which we will discuss in the general discussion.

In the memory task, reaction times revealed that when the response key set was up-yes, arrows presented at the top of the screen decreased reaction times for negative test words. When test words were positive, positive memory words decreased participants’ reaction times. Positive memory words even facilitated participants’ responses when arrows pointed up and test words were negative. These findings indicated that arrows presented at the top of the screen and positive words facilitated memory task performance. When the response key set was up-no, the interaction between memory and test word was also significant. Positive memory words facilitated participants’ responses when test words were positive, which further indicated that positive memory words affected memory task greater than negative words. Accuracy had a similar pattern to reaction time. The finding that positive memory words had a greater effect on the following tasks might be explained by positive bias. Previous studies have found that normal participants responded faster to positive facial expressions, and oriented more quickly toward positive faces, which might reflect a higher-level asymmetry in processing positive and negative emotions [32], [33].

## General Discussion

The ERP experiment found that negative words induced enhanced P200 amplitude compared to positive words when spatial cues were presented at the bottom of the screen; top spatial cues elicited larger P200 amplitudes than did bottom spatial cues when participants kept positive words in mind. The behavioral experiment revealed that positive words facilitated the discrimination of upper arrows. In summary, we found significant P200 amplitude differences and a facilitation effect under metaphorical congruency conditions.

The P200 might be related to attention allocation. Larger P200 amplitude indicates greater attentional resources allocated to cognition [34], [35]. The P200 potentials at prefrontal and central sites were mainly related to rapid awareness of stimulus features [36]. In another study, target stimuli in visual feature selection and spatial selection induced the P200 in prefrontal electrodes [37]. The P200 potentials in frontal and central electrodes were sensitive to stimuli that contained target stimuli or their features [38]. Therefore, the P200 might constitute a valid electrophysiological substrate for attention allocation and target stimuli detection.

Further, Gole et al. [39] suggest that the P200 reflects early automatic attention allocation for threatening information or warning cues. In word processing, Ma et al. [40] postulated that the P200 is an attention-related component and that enhanced P200 amplitude indicates the allocation of attentional resources to evolutionarily significant stimuli. For example, warning signal words elicited an enhanced P200. Hence, the P200 suggested early automatic and rapid processing of potential hazards in warning words. Junhong et al. [41] found that unattended fearful faces elicited enhanced P200 amplitude, as measured at frontal and central electrode sites. In their experiment, participants judged the structure (low cognitive load) or tone (high cognitive load) of characters while exposed to unattended faces. The enhanced P200 was only observed in the low cognitive load condition. Hence, the P200 might be an index of automatic attention and automatic detection of emotions.

In the current experiment, participants retained affective words during the elicitation of the P200. However, the enhanced P200 in our experiment was not moderated by valence alone. It was moderated by the metaphorical congruency effect, which contained both valence and spatial information. Since the P200 was found at the frontal and central electrodes during valence-space metaphor processing, the present P200 might also reflect automatic attention allocation.

Lijffijt and colleagues [42], [43] acknowledged that the P200 might relate to the allocation of attention and the initial awareness of a stimulus. Yun et al. [44] found that PTSD participants who had experienced an earthquake disaster elicited an enhanced P200 amplitude when exposed to earthquake-related words. The enhanced P200 might reflect extra attentional resource allocation to earthquake-related words. Hence, the P200 might be associated with automatic attention selection and perceptual analysis of stimuli. The P200 in our experiment was elicited by spatial cues that did not contain any affective information. However, affective words that were kept in mind might also affect the detection of these cues because of the valence-space metaphor. The P200 component might reflect the allocation of attention and the initial awareness of cues, while these processes were moderated by affective words.

Ferreira-Santos et al. [45] summarized some P200 findings and found that the P200 was related to not only selective attention or stimulus encoding processes, but also the process of making comparisons between predicted and actual perceived environmental states. Furthermore, the P200 might actually be two separate components: the exogenous P150 (120–200 ms) and the endogenous P250 (220–280 ms). In our experiment, the P200 component was chiefly elicited after 200 ms, and it was moderated by the metaphorical congruency effect. Hence, the P200 in our experiment may have been the endogenous P200. It is plausible that processing affective words precipitates activation of spatial information and subsequently shifts attention to corresponding locations.

Keeping affective words in mind influenced ERP differences around 200 ms. According to previous studies, early ERP components might reflect attention shifting and other automatic cognitive processing [46]. Therefore, in the current experiment, the P200 indicates that affective words might influence the following spatial cue detection tasks by arousing attention shifting. Attention shifting may have thus resulted in P200 amplitude differences in four different trial types.

However, these ERP findings might also be explained by response preparation. When participants kept affective words in mind, participants might allocate their attention to corresponding locations, and simultaneously prepare for response. Although participants were asked to respond after a blank screen, they still require response preparation. From this view, response preparation might also explain the findings from Experiment 1. Hence, we conducted Experiment 2 in order to counterbalance response preparation.

ERP results demonstrated metaphor congruency effects, which might also be explained by polarity correspondence. Polarity correspondence indicates that + polar, but not – polar, endpoints of dimensions facilitate category processing [47]. In the vertical dimension, up and down are generally considered + polar and – polar, respectively. In the valence dimension, positive and negative are generally considered + polar and – polar, respectively. Polarity correspondence holds that participants’ reaction times are faster for + polar words presented on the top of the screen than for + polar words presented on the bottom of the screen. Further, there would be no difference between – polar words that were presented at the bottom or top. In our experiment, we only found significant results when memory words were positive (+ polar), and spatial cues were presented at the bottom of the screen (– polar). If we use the valence dimension to distinguish our results, our findings might support polarity correspondence. However, if we use the vertical dimension to distinguish our results, our findings could not be explained by polarity correspondence, because we only found a significant difference when spatial cues were presented at the bottom (– polar). However, our results were asymmetric, and polarity correspondence could partially explain the results in Experiment 1.

Behavioral data from the ERP experiment did not reveal any significant effects related to our main hypothesis, perhaps because the behavioral response was an offline response due to the experimental design. Hence, we conducted a separate behavioral experiment. Independent behavioral data revealed a facilitative effect in the metaphorical congruency conditions, similar to the ERP findings. Keeping affective words in mind affected processing of arrow location but not discrimination of arrow direction. It is possible that keeping affective words in mind activated spatial information that facilitated the locating of arrows. However, this effect was only observed when participants kept positive words in mind, which was consistent with polarity correspondence. According to polarity correspondence, participants only had faster reaction times while + polar words were presented up than for + polar words were presented down. On the contrary, such an effect would not be found in – polar words. In Experiment 2, we only found metaphor congruency effects when memory words were positive as a finding that could be accounted for by polarity correspondence.

This finding was similar to previous studies. Meier and Robinson [1] found that discriminations for letters in the up position were faster after positive priming words, while discriminations for letters in the down position were faster after negative priming words (study 2). Gozli et al. [17] found a similar facilitation effect using detection and discrimination tasks. Differences between the current experiment and previous experiments were the priming words and the discrimination task. Previous studies asked participants to judge priming words prior to or simultaneous with the discrimination task, while the current study asked participants to keep valence words in mind during the discrimination task. In the current experiments, we also found a metaphorical congruency effect when valence words were kept in mind. In the discrimination task of Experiment 2, we tested the location and direction of arrows simultaneously, which might help us distinguish attention allocation and response preparation. Our results only found a metaphorical congruency effect between valence word and arrow location, which might support the attention allocation account.

The results in Experiment 2 could also be explained by other theories. In Experiment 2, arrows with different directions were presented on the top or bottom of the screen. Participants distinguished arrow direction, and ignored arrow location. Hence, arrow location could attract participants’ attention, and did not require participants’ responses. On the contrary, arrow direction did require participants’ responses. If processing valence words mainly resulted in attention allocation, the metaphor congruency effect would be found between valence words and arrow location, which was supported by our results. However, response preparation might also participate in such a metaphor congruency effect. In addition, we found a spatial Stroop-like effect between arrow location and arrow direction, which indicated that participants might automatically activate response preparation. When they detected the locations of arrows, they also prepared to press consistent keys to make responses (e.g., positive words were consistent with upper keys). In Experiment 2, when participants detected top or bottom arrow locations they might automatically prepare to press upper or lower keys, respectively. However, we did not find the interaction between memory words and arrow direction. The alternative explanation, response preparation, might not be supported directly by our results. Above all, the results of Experiment 2 seem to mainly support attention allocation, while these results might be also affected by response preparation.

The results of the current experiments were independent of the response key set. In two experiments, participants memorized one affective word (negative or positive) at the beginning of each trial. At the end of each trial, they saw one word with question marks (“?Negative?” or “?Positive?”) on the screen. They judged whether the word correctly described the memorized word. They used a keyboard to respond “Yes” or “No.” We counterbalanced the pressing keys, half pressed “up-yes” or “down-no” and another half pressed “up-no” or “down-yes.” Participants only judged the location of spatial cue (Experiment 1; top or bottom) or direction of arrow (Experiment 2; up vs. down) and whether the description was correct (yes vs. no). Hence, participants did not need to judge the valence of affective words using response keys. This configuration of response keys avoided the congruent (positive-up) and incongruent (positive-down) valence-space response code, as some studies have found positive was related with up and negative was related with down [1]. Thus, the results of the current experiments did not depend on the response key set and likely reflected the true effects of affective words on spatial locating.

Why might keeping affective words in mind affect spatial locating? Embodied cognition stipulates that abstract concepts are grounded in concrete concepts, that is, they form metaphors [15]. For example, spatial information and body orientation information are commonly used in processing abstract concepts. People rely on spatial information to understand abstract concepts, such as time. In daily life, people often say a long vacation or a short concert [11]. Studies have indicated that abstract concepts rely on spatial information in conceptual processing, such as weight, power, number, quality, temperature, and so forth [7], [25], [48], [49]. According to embodied cognition, negative words establish connections with bottom space and positive words establish connections with top space. Because of the abstraction of affective words, people need perceptual information to engage in language processing [50]. When they understand affective words, they also activate spatial information to help them understand these words. When spatial information is activated, it could facilitate performance on the following spatial task.

Do affective words shift attention automatically? In the view of embodied cognition, the representations of affective words are grounded in sensorimotor information. As a necessary element in representation, sensorimotor information is also automatically activated while affective words are used in order to improve language processing [19], [51]. However, in this experiment, we cannot conclude that spatial information was activated automatically. Although the P200 might be an index for automatic attention allocation, and previous studies have found that spatial attention is automatically activated in cognitive processing, the relationship between the P200 and cognitive processing remains unclear [52], [53]. The present findings could not directly support the hypothesis that affective words shift attention automatically. However, while the task in our studies did not allow for participants to connect affective words with spatial cues and the enhanced P200 component, affective words might automatically shift attention.

The results also indicated that people used sensorimotor information to understand emotion. In this experiment, keeping affective words in mind activated spatial information, which is one type of sensorimotor information, and spatial information might influence participants to shift their attention to corresponding locations. Hence, for the same spatial cues, affective words enhanced P200 at frontal and central sites under metaphorical congruency conditions. Previous research has found that the P200 potential is more pronounced in attentive direction than in undistinguished direction [54]. Therefore, the P200 component might indicate that participants pay more attention to metaphorical congruency locations. Behavioral data also demonstrated the metaphorical congruency effect. Two experiments provided evidence that participants used metaphor to understand affective words, and further, that metaphor may be caused by attention shifting. These findings are also consistent with previous studies, which have found that when one changed one’s bodily states, emotion was subsequently changed [55], [56]. The current study found the metaphorical congruency effect that may be caused by the activation of spatial information. Barsalou [4] and Niedenthal [7] believe that affective concepts are based on sensorimotor information, such as spatial information. Our findings further suggest that sensorimotor information participated in the understanding of emotion in affective words even when participants kept affective words in mind, which might also provide perspective for emotional research.

However, several questions remain for further research to address. First, though metaphor might be caused by attention shifting, whether the metaphor is based on primary visual areas in the brain remains unknown. While primary visual areas constitute the base of attention, if we could demonstrate that primary visual areas participate in the valence-space metaphor, we could more confidently state that the valence-space metaphor is automatic. Second, embodied effects might only appear in the left visual field [9], [10]. Is metaphor activated solely in the left visual field? This question must be addressed. Third, conceptual representations not only contain spatial information but also other sensorimotor information. How are these representations used in conceptual processing? Future studies should address this question.

In summary, the inner mechanisms of the valence-space metaphor were tested in this experiment. These findings provide some new perspectives for embodied cognition, namely, that affective words might activate spatial information in word processing, known as the valence-space metaphor. The valence-space metaphor might be caused by attention shifting when affective words activate spatial information. These findings also provide some new perspectives for understanding emotion in affective words. Emotional processing might be grounded in sensorimotor information, such as spatial information, and this information is subsequently used to understand emotion.
